# An atypical presentation of orthostatic hypotension and falls in an older adult

**DOI:** 10.29045/14784726.2022.03.6.4.41

**Published:** 2022-03-01

**Authors:** Steve Thoburn, Steve Cremin, Mark Holland

**Affiliations:** North West Ambulance Service NHS Trust; University of Bolton; North West Ambulance Service NHS Trust; University of Bolton

**Keywords:** accidental falls, emergency medical technicians, orthostatic hypotension

## Abstract

**Introduction:**

Falls are a significant cause of morbidity and mortality in older adults. Orthostatic hypotension (OH) is very common in this cohort of patients and is a significant risk for falls and associated injuries. We present the case of an 89-year-old female who fell at home, witnessed by her husband. OH was identified during the clinical assessment and considered to be the predominant contributing factor, although the clinical presentation was not associated with classical symptoms.

**Case presentation:**

The patient lost balance while turning away from the kitchen sink; she noted some instability due to a complaint of generalised weakness in both of her legs. No acute medical illness or traumatic injury was identified. A comprehensive history was obtained that identified multiple intrinsic and extrinsic risk factors for falling. The cardiovascular examination was unremarkable except for OH, with a pronounced reduction in systolic blood pressure of 34 mmHg at the three-minute interval and which reproduced some generalised weaknesses in the patient’s legs and slight instability. Although classical OH symptoms were not identified, this was considered to be the predominant factor contributing to the fall. A series of recommendations was made to primary and community-based care teams based upon a rapid holistic review; this included a recommendation to review the patient’s dual antihypertensive therapy.

**Conclusion:**

It is widely known that OH is a significant risk factor for falls, but asymptomatic or atypical presentations can make diagnosis challenging. Using the correct technique to measure a lying and standing blood pressure, as defined by the Royal College of Physicians, is crucial for accurate diagnosis and subsequent management. Ambulance clinicians are ideally placed to undertake this quick and non-invasive assessment to identify OH in patients that have fallen.

## Background

Falls are a significant cause of morbidity and mortality in older adults; it is estimated that up to 30% of adults ≥ 65 years old and 50% over the age of 80 years old experience at least one fall annually (National Institute for Health and Care Excellence [NICE], 2013). The financial impact of falls on the NHS is estimated at £2.3 billion per year (NICE, 2013). Orthostatic hypotension (OH) is frequently cited as a significant risk factor for falls and syncope (Freeman et al., 2020; Juraschek et al., 2017), increasing the risk of trauma-induced fractures, head injury and hospitalisation (Juraschek et al., 2017; Shibao et al., 2007). It is defined as a sustained reduction in systolic blood pressure (BP) of ≥ 20 mmHg, or ≥ 10 mmHg drop in diastolic BP or a decrease in systolic BP < 90 mmHg within three minutes of standing (Brignole et al., 2018). We present the case of an 89-year-old woman who fell at home in the presence of her husband. OH was identified during the clinical assessment and considered to be the predominant contributing factor.

## Case presentation

### History and initial assessment

The patient stated that she lost balance and fell while turning away from the sink in her kitchen. The event was witnessed by her husband who corroborated the patient’s account. The patient did not experience transient loss of consciousness (TLoC) or any red flag prodromal symptoms; however, she did state that prior to losing balance both of her legs felt weak. The patient’s husband immediately called for an ambulance that attended within 30 minutes of the fall.

The patient was found in the supine position on a firm, carpeted floor. A primary survey was conducted which revealed no significant physiological abnormality (Table 1) in the supine position; her NEWS2 score was 1. No traumatic injury to the axial or appendicular skeleton was identified upon physical examination; the patient was not in pain and there was no bony tenderness. No skin integrity concerns were identified. She reported no new symptoms since the fall.

**Table 1.** Initial observations.

Observation	Reading	Units
Heart rate (lying vs. seated vs. standing)	93 lying, 98 seated, 107 standing	Beats per minute
Respiratory rate	20	Breaths per minute
BP (lying)	147/86	mmHg
BP (standing 1 min)	130/84	mmHg
BP (standing 3 mins)	113/90	mmHg
Oxygen saturation	96	% (on air)
Temperature	37.4	°C
Blood sugar	7.8	mmol/L
GCS	15	
Delirium score (4AT)	1 (possible cognitive impairment)	
Pain score*	0	
Clinical frailty scale score	6 (moderately frail)	

*Numerical pain rating scale.

BP = blood pressure; GCS = Glasgow Coma Scale.

Once upright, the patient was able to weight-bare and mobilise normally. There was no evidence of restricted joint movement beyond what was normal for her, although gait speed was slow, taking her eight seconds to walk four metres. The patient stated that she had recently been well and a review of systems inquiry was unremarkable.

### Medical history

Further history-taking revealed a previous medical history of hypertension, glaucoma, cervical spondylosis, cholelithiasis, osteoarthritis, as well as four falls in the last 12 months. Her husband also stated that her memory was declining, but she had not seen her GP for some time. The patient had a DNACPR. The patient’s prescribed medication and dose is provided within Table 2.

**Table 2.** Prescribed medication and dose.

Medication	Dose
Bisoprolol Fumarate	10 mg once daily
Felodipine	10 mg once daily
Simvastatin	40 mg once daily
Codeine phosphate	15 mg four times daily if required
Ibuprofen 10% gel	Up to four times daily if required
Alphagan eye drops 0.2%	
Ganfort eye drops	

### Psychosocial history

Due to her increasing frailty, the patient was now considered housebound; she resided with her husband in a bungalow. They were not in receipt of any formal care. Their only daughter lived three hours away; however, she visited regularly and was actively involved in promoting their health, safety and independence. The patient had received some mobility aids and home adaptions, including a personal alarm system, but she had not been reviewed for some time as COVID-19 had impacted upon the availability and extent of community rehabilitation interventions.

## Physical examination

### Cardiovascular examination

A cardiovascular examination was conducted after excluding any acute illness or injury, as it is well recognised that many falls are in fact the consequence of an unrecognised syncopal or presyncopal episode (O’Brien & Kenny, 2014). The examination was unremarkable; the findings are presented in Table 3. However, an assessment of lying and standing BP (LSBP) conducted in line with Royal College of Physicians (RCP, 2017) guidance revealed a pronounced reduction is systolic BP at the three-minute interval (see Table 1). This was not associated with any classical prodromal symptoms of light-headedness, dizziness or impending blackout, but of a vague complaint of generalised leg weakness; the patient also appeared more unsteady and expressed a desire to sit down. These clinical findings did not present until close to the three-minute interval point.

**Table 3.** Cardiovascular examination findings.

Skin	No evidence of pallor, cyanosis or diaphoresis
Mucous membranes	Moist, no tongue furrows
Jugular venous pressure	Not elevated
Heart sounds	SI – SII – 0. No murmur, clicks or rubs
Lung bases	Vesicular air entry bilaterally, no crackles
Peripheral pitting oedema	No evidence of pitting oedema in the ankles / lower legs
Lower leg	No evidence of unilateral calf swelling, tenderness, heat or erythema

A 12-lead ECG was also conducted which revealed no acute changes or conduction abnormalities. Sinus rhythm was recorded while seated, and a slight sinus tachycardia identified upon standing (Table 1).

## Further assessment

The 4AT score was used to exclude delirium, but the outcome was suggestive of cognitive impairment. Through discussion with both the patient and her husband it became apparent that her cognition had been slowly declining over a timeframe of 12 months. Table 4 provides an overview of the differential diagnoses considered, but excluded through clinical reasoning. The patient had noticed a reduction in general strength, reduced exercise tolerance, further reduction in gait speed, increased frequency of falls and a fear of further falls. These concerns are well recognised as risk factors for perpetuating frailty, the progression towards disability and dependence and the risk of further falls (Rahman, 2019).

**Table 4.** Differential diagnoses.

Differential diagnosis	Clinical reasoning process
TIA or stroke	A collateral history from the husband and the patient’s assessment revealed no evidence to suggest an acute neurovascular event; there was no dysarthria or dysphasia, no new neurological findings on examination and the patient was FAST negative.
Infection	There was no history of fever or rigors, and no symptoms to suggest an infection from a common source, such as lower respiratory tract, skin or urinary tract. There was no recent acute illness and no evidence of delirium (4AT score of 1). In the absence of any other evidence for an infection, the isolated tympanic temperature of 37.4 C was considered to be of low clinical significance and within accepted limits, particularly for a warm ambient environment.
Cardiac syncope (arrhythmogenic and structural)	No evidence of an arrythmia was found, although recurrent symptoms following treatment of the OH would require further investigation for a paroxysmal abnormality. Importantly, in an older patient with a fall, clinical examination showed no evidence of a heart murmur to suggest a valvular abnormality, especially aortic stenosis.
Neurally mediated syncope (vasovagal, situational)	This was deemed unlikely in the absence of classical prodromal symptoms and no specific trigger.
Volume depletion (dehydration)	Cardiovascular examination revealed no concerns regarding volume status. The patient reported regular urine output, light yellow in colour. There was no recent vomiting or diarrhoea. Good oral intake of fluids was noted.
Metabolic (hypoglycaemia)	A capillary blood glucose was measured to exclude hypoglycaemia as a cause of the fall. The modestly elevated glucose of 7.8 mmol/L is within normal limits for an adult who has recently eaten (approximately one hour prior to the event in this case) and was not a concern for new onset diabetes mellitus.

OH = orthostatic hypotension; TIA = transient ischaemic attack.

### Management

OH was diagnosed based upon the criteria outlined by the Joint Royal Colleges Ambulance Liaison Committee & Association of Ambulance Chief Executives (JRCALC & AACE, 2019). The significant reduction in systolic BP was accompanied by less classical symptoms of generalised leg weakness and unsteadiness. The medication history revealed dual antihypertensive therapy with bisoprolol and felodipine. The orthostatic drop was considered to be the most significant factor contributing to the patient’s fall. However, multiple other risk factors were identified and, in line with guidance from the British Geriatrics Society (BGS, 2014), generating a problem list (Table 5) enabled a patient-centred discussion about concerns and goals, which subsequently led to shared decision-making about onward referrals and interventions. The patient was referred to their GP for a review by the Housebound Frailty Team and Community Rehabilitation Service. A recommendation was made that this should include a structured medication review focusing upon the dual antihypertensive therapy.

**Table 5.** Patient-specific problem list.

Category	Patient-specific problem
Physical	Arthritis causing chronic pain. Postural hypotension, systolic dropped by 34 mmHg upon standing. Falls becoming more frequent.
Socioeconomic/environmental	Difficulty with bathroom and kitchen.
Functional	Support and dependence for instrumental activities of daily living from husband.
Mobility balance	Poor balance, slow gait speed. Housebound, limited physical activity, easily exhausted, reduced strength.
Psychological/mental	Cognition – intermittently confused, forgetful. Fear of falling.
Medication review	Dual antihypertensive treatment – possible cause of postural instability and falls.

## Follow-up and outcomes

The patient was visited by the Housebound Frailty Team and had a reduction in her felodipine dose to 5 mg once daily. An occupational therapist and physiotherapist from the Community Rehabilitation Service were involved in the completion of a multi-factorial falls risk assessment. The patient subsequently underwent community rehabilitation, which included strength and balance interventions. There have been no further falls reported in the six months following the case report incident.

## Discussion

### Orthostatic hypotension

Falls in older adults often occur as a result of a complex interplay between both intrinsic and extrinsic factors (Anderson, 2008; JRCALC & AACE, 2019). Multiple risk factors were identified in this case study, but clinical reasoning determined that OH was the predominant risk factor and was diagnosed by a marked drop in systolic BP greater than 20 mmHg upon standing (RCP, 2017). It is well recognised that OH can contribute to postural instability, falls and syncope (Brignole et al., 2018; O’Brien & Kenny, 2014); however, its exact prevalence is difficult to determine. In community-dwelling adults, estimates of prevalence are between 5% and 30% for adults ≥ 65 years old; this increases to 60% for individuals with Parkinson’s disease and up to 70% in nursing home residents (Freeman et al., 2011; NICE, 2015).

### Atypical presentations

Distinguishing syncope from falls in older adults is complex (O’Brien & Kenny, 2014). The diagnostic process is further complicated by the impact of transient cerebral hypoperfusion, which may induce amnesia of the event, leading to patient confabulation of ‘a trip’ to rationalise the fall (O’Dwyer et al., 2011; Parry et al., 2005). OH is classically associated with the symptoms of light-headedness, dizziness and impending blackout, but emerging empirical data suggest asymptomatic presentations and less classical symptoms are very common in older adults which can ultimately impact upon accurate and timely recognition of the condition (Benvenuto & Krakoff, 2011; Freeman et al., 2020). The findings of Freeman et al. (2020) were applicable to the whole cohort of participants, but patients with multiple system atrophy, Parkinson’s disease and Lewy body dementia were shown to be asymptomatic at even lower BPs. The pathophysiological process to explain this phenomenon is not yet understood, although hypotheses have been put forward that it may be due to: reduced sensory perception of OH, the impact of cerebral hypoperfusion, physiological habituation and innate variations in autoregulatory mechanisms (Freeman et al., 2020). Consequently, it has been postulated that the absence of prodromal symptoms, which act as a protective warning mechanism, may increase the risk of falls and subsequent injuries (Freeman et al., 2020). The absence of classical prodromal symptoms, as evident in this case, may falsely reassure clinicians that OH is not implicated in the cause of falls. Based upon the developing evidence base it would be advisable, in the absence of a clearly distinguishable extrinsic factor, to assess for OH.

### Orthostatic hypotension management

Multiple classes of medication induce OH; calcium channel blockers, as prescribed to this patient, are known to cause OH (Juraschek et al., 2018). The management of OH frequently involves a multi-faceted yet balanced approach to raise standing BP, reduce orthostatic symptoms and improve functional status without elevating supine BP. In the absence of an obvious acute medical presentation precipitating OH, the initial step is to identify any possible medications that may be the cause, and either reduce or discontinue them (Figueroa et al., 2010).

### Lying and standing blood pressure measurement

**Figure 1:**
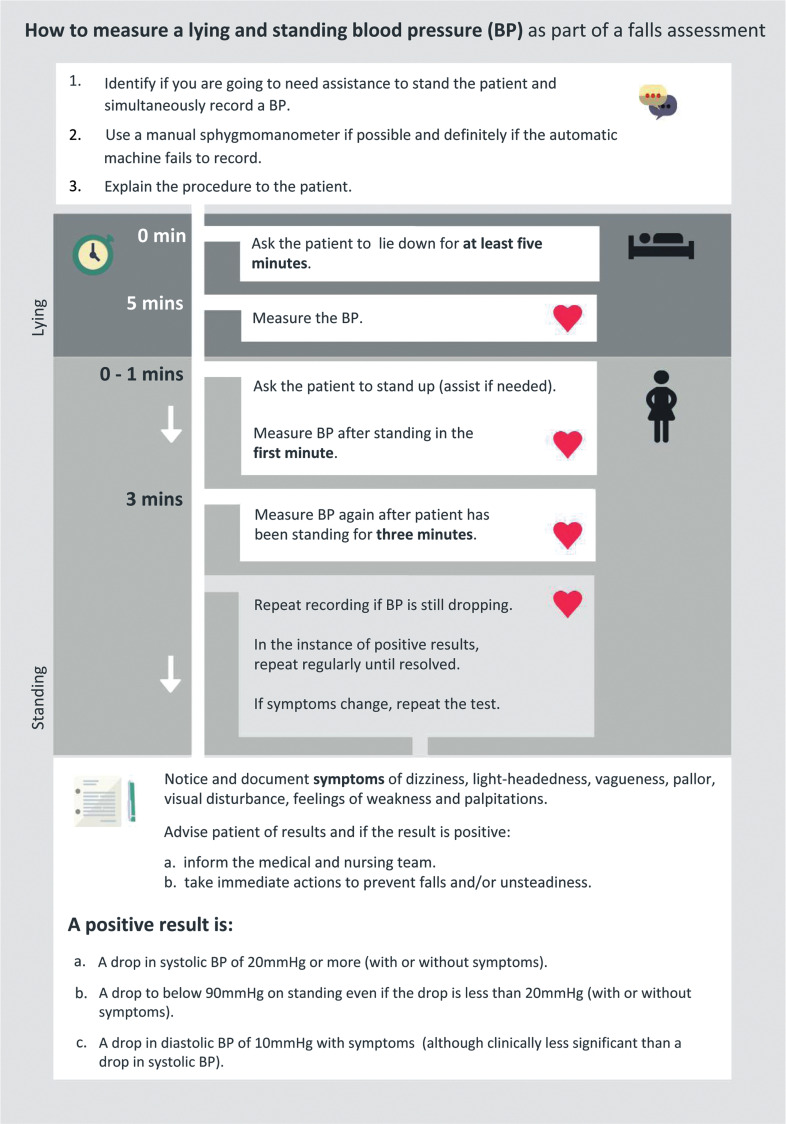
Figure 1. How to measure a lying and standing blood pressure as part of a falls assessment.

In order to identify patients that may require intervention to reduce the impact of OH, it is of critical importance to accurately make the diagnosis using an accurate measurement procedure. In the 2015 national audit of inpatient falls (NAIF), Vasilakis et al. (2015) identified that only 16% of 4846 eligible patients had an LSBP recorded by their third day in hospital. A review of the literature, which focused upon clinician knowledge and understanding of the correct OH measurement procedure, identified that in general, participating clinicians had poor knowledge of the OH measurement process, and the procedure they utilised to physically measure LSBP was inaccurate to diagnose OH (Barsaiyan & Mildner, 2014; O’Riordan et al., 2017; Vloet et al., 2002). The participants were predominantly nurses, and some of the studies were dated so extrapolation of the findings to ambulance clinicians requires caution. However, analysis of the literature has unearthed practice-based issues that may extend to allied professions such as paramedics and emergency medical technicians. In view of this literature, further exploration of pre-hospital practice would be reasonable to ensure compliance with best practice. Furthermore, the authors identified that many participants did not utilise the correct timings, procedure or sequence of positions when measuring LSBPs. Variations in position, such as sitting to standing BPs, are not sufficiently accurate enough to diagnose OH with a high degree of sensitivity and specificity (Cooke et al., 2009; Shaw et al., 2017). Therefore no recommendations, other than to follow the positions and measurement procedure in current guidance by the RCP (2017), can be justifiably made to accurately measure OH (Figure 1).

©2017 Royal College of Physicians. Reproduced with permission.

## Conclusion

Falls in older adults are often multi-faceted in origin, but this case study highlights that OH is a significant risk factor for falls. Asymptomatic cases or those with atypical prodromal symptoms are encountered more frequently than expected, which emphasises the importance of a thorough history, physical examination and correct LSBP measurement to accurately diagnose OH. Falls are one of the most common 999 presentations; therefore, ambulance clinicians are ideally positioned to undertake this quick and non-invasive assessment, to identify OH and subsequently reduce the risk of further falls. Furthermore, referrals into falls assessment services enable multi-factorial falls risk assessments to be conducted by specialist clinicians that are both holistic and patient-centred, in order to help older adults maintain independence and a functional health status.

## Take-home points

- OH is a significant risk factor for falls and is very common in older adults.
- OH can occur in the absence of symptoms, or they may be subtle or non-specific.
- The accurate diagnosis of OH is dependent upon the correct LSBP measurement process as defined by the Royal College of Physicians.
- Ambulance clinicians are ideally positioned in the pre-hospital setting to undertake this quick and non-invasive assessment to identify OH as a risk factor for falls.

## Acknowledgements

We thank the patient for providing consent to publish this case report. We also express our thanks to the Royal College of Physicians for granting us permission to use the lying and standing blood pressure timeline graphic.

## Author contributions

ST and SC attended the patient in the case study. ST drafted the initial version. All authors contributed to revision and agreed on the final version of the manuscript. ST acts as the guarantor for this article.

## Conflict of interest

None declared.

## Funding

None.
